# A Study on the Influencing Factors of Consumers' Purchase Intention During Livestreaming e-Commerce: The Mediating Effect of Emotion

**DOI:** 10.3389/fpsyg.2022.903023

**Published:** 2022-05-09

**Authors:** Rong Zhou, Lei Tong

**Affiliations:** ^1^Department of Development Studies, Faculty of Business and Economics, University of Malaya, Kuala Lumpur, Malaysia; ^2^Faculty of Tourism Management, Wuhan Business University, Wuhan, China

**Keywords:** live streaming e-commerce, emotional marketing, emotional trust, perceived emotional value, purchase intention

## Abstract

With the deep popularity of mobile Internet, the “eyeball economy” is more active than ever. Driven by powerful modern media, livestreaming, as a new form of attracting public attention to obtain economic benefits, is worth studying its influence path on consumers. Based on the technology acceptance model and the mediating effect of emotion, this study constructs the consumer influencing factor model of livestreaming e-commerce. The research model and related hypotheses are verified by SPSS and linear multiple regression models. The research found that emotional trust and perceived emotional value could be regarded as mediating variables to stimulate consumers' purchase intention in livestreaming e-commerce. They have a full mediating effect on product and atmosphere and a partial mediating effect on homogeneity and promotion, which identifies that online celebrity's homogeneity, and sales promotion could influence consumers' purchase intention through the partial mediating role of emotional trust and perceived emotional value, while product and atmosphere induced by emotional contagion could exert influence on consumers' purchase intention through the full mediating effect of emotional trust and perceived emotional value.

## Introduction

E-commerce live broadcast (livestreaming e-commerce) is a type of e-commerce in which live broadcast is used as a marketing channel (Kang et al., 2021; Xie C. et al., 2022). It is the result of the two-way integration of live broadcast and e-commerce in the digital age (Wongkitrungrueng and Assarut, 2020; Zhou et al., 2021). E-commerce live broadcast uses live broadcast to reconstruct the three elements of “people, goods, and field,” but its core is still e-commerce (Xue et al., 2020; Lu and Chen, 2021). Compared with traditional e-commerce, e-commerce live broadcast has the advantages of high interaction and high conversion rate (Xu et al., 2021). The live broadcast e-commerce industry chain is made up of the supply side, the platform side, and the consumers. The upstream supply side primarily consists of commodity suppliers (e.g., manufacturers, brands, distributors, and origin) (You, 2020); the midstream primarily consists of live broadcast service providers, channel platforms (e.g., e-commerce platforms, content platforms, and social platforms) (Liu et al., 2021), and anchors (e.g., Internet celebrities, star artists, entrepreneurs, and other anchors); and the downstream demand side primarily consists of consumption (Maier and Wieringa, 2021). In the traditional centralized e-commerce model, the platform is the center of transactions and communication between brands, merchants, and consumers, and it has tight control over flow, transaction data, and customer relationships (Yue et al., 2019; Varadarajan et al., 2021; Peruchi et al., 2022). With the development of socialization, containerization, and decentralization of e-commerce, the platform's strong control over merchants and consumers has gradually eroded (Li et al., 2021), allowing brands, merchants, and consumers to have direct contact with consumers and make sales conversion more efficient and cost-effective (Gielens and Steenkamp, 2019; Wedel et al., 2020). From flow acquisition to conversion operations, the value of private domain flow is increasing.

As the flow cost of centralized platforms continues to rise, the platform's control over flow has weakened to varying degrees (Loux et al., 2020; Wagner et al., 2021). On the one hand, the centralized e-commerce platform began to receive external flow through technical cooperation, investment and acquisition, and so on (Cui et al., 2017; Malacina et al., 2022); on the other hand, the platform's flow control has tightened further, causing more flow to be concentrated on head companies and clearing out mid- and long-tail merchants (Chiu and Wong, 2021; Gao et al., 2022). Under these conditions, the combination of small- and medium-sized businesses and private domain flow becomes even more important (Jun et al., 2020). When the flow is plentiful, a significant amount of free or low-cost public domain flow can be converted into private domain flow of brands and merchants, with the private domain flow of merchants being deposited on the platform and becoming the platform's public domain flow (Xie X. et al., 2022). When the flow dividend falls, however, the platform's demand for public domain flow control and monetization rises, amplifying the conflict between public and private domain flow (Fu et al., 2022). Second, private domains are not a brand-new idea. Address books and emails were all examples of private domain flow before social networking became popular (Cozzolino et al., 2021). The connotation of private domain flow has been further extended with the emergence of social platforms, such as WeChat and Weibo.

On the one hand, many brands and merchants have amassed a portion of the initial “fans” in the traditional e-commerce model, but users of the centralized platform belong to the platform, and their relationship with the brand merchants is not close (Phang et al., 2013; Ertz and Boily, 2019). Importing users into their own platforms is the only way to achieve controllable private domain flow (e.g., independent app, WeChat group, official account, personal account, and corporate WeChat). Brand merchants, on the other hand, must pay for public domain flow pool exposure, which will continue to rise in price (Wong and Ngai, 2021; Zheng et al., 2022). In contrast, while merchants must also operate and maintain private domain flow, the cost of acquiring customers is relatively low and long term (Peck et al., 2013; Kumar et al., 2017). Finally, as more people become aware, brands and merchants will be able to establish private domain flow pools, allowing them to expand their business. Livestreaming e-commerce emerges at a historic moment and is worth studying its influence path on consumers. Based on the technology acceptance model and the mediating role of emotion, this study constructs the consumer influencing factor model of livestreaming e-commerce. The research model and related hypotheses are verified by SPSS and linear multiple regression models.

In conclusion, the main theoretical contributions of this study are as follows. Although there are many literatures on emotional marketing and livestreaming e-commerce, this study is the first to use emotional trust and perceived emotional value as mediating variables to study the effect of emotion on consumers' purchase intention in livestreaming e-commerce. In addition, although there are many literatures on opinion leader, marketing mix, and emotional contagion previously, this study is the first to take the characteristics of online opinion leaders, product and promotion, as well as atmosphere induced by emotional contagion as independent variables, plus emotional trust and perceived emotional value as intermediary variables, to study how these variables influence consumers' purchase intention through the mediating effect of emotion in livestreaming e-commerce.

## Literature Review and Research Hypotheses

### Emotional Marketing, Emotional Trust, and Perceived Emotional Value

With the continuous development of the digital economy, plus traditional marketing activities could not excite consumers, emotional marketing becomes a key explanatory construct in the field of consumer behavior (Honea and Dahl, 2005). Previous studies have shown that emotional reaction could be triggered by the physical purchase environment (Yoo et al., 1998), the virtual shopping environment (Meng et al., 2021), product displayed (Oliver, 1994), price promotion (Aydinli et al., 2014), and emotional attachment to online celebrities (Ladhari et al., 2020). These emotional responses have been shown to influence attitudes, evaluations, and behaviors (Honea and Dahl, 2005).

Emotional trust is the trust formed between the trusting party and the trusted party on an emotional basis; it is concerned about the welfare of the trusting party and takes full account of the trusting party's purpose and intentions; it relies on good communication (Chua et al., 2008). Emotional trust is based on mutual interaction and attraction; frequent communication and exchange between individuals deepen the relationship over time and shows concern for the welfare of the trusted person (Mian and Hattab, 2013). Consumers' perceived trust in online retailers influences their emotional trust, which in turn enhances purchase intentions (Zhang et al., 2014). Consumers have emotional trust in the brand community, which increases their willingness to buy the brand (Habibi et al., 2014). Emotional trust will influence consumer purchase intentions (Awad and Ragowsky, 2008). Consumers are more likely to choose products recommended by trusted opinion leaders because their emotional trust influences their purchasing decisions (Koufaris and Hampton-Sosa, 2004).

Perceived emotional value is associated with the feelings of affection, love, connection, and passion. It is recognized that consumers will perceive the emotional value to products, promotions, environment, brands, and celebrities (Thomson, 2006; Dwivedi et al., 2018). Emotional value measures the perceived utility that consumers associate with a product or service's ability to elicit an emotional or affective state (Sheth et al., 1991). Emotional value is influenced by the benefits consumers derive from the goods or service (Sweeney and Soutar, 2001). Rational and emotional factors associated with a product or service play an important role in all purchase decisions (Mackay and Mackay, 1999). Therefore, this study aims to study the mediating effect of emotion in livestreaming e-commerce. In this article, emotion is divided into two mediating variables, namely, emotional trust and perceived emotional value.

### Online Celebrity

Opinion leaders have a deeper understanding of products and services, can provide information and advice to others, and have a significant influence on the attitudes of followers (Rogers and Cartano, 1962; Stern and Gould, 1988), so online celebrities can be considered network opinion leaders to some extent. Network anchors, unlike traditional opinion leaders, regularly release self-created topics on social media or e-commerce platforms *via* network live broadcasts, videos, photos, blogs, and other means to influence their followers (Mcquarrie et al., 2013; Khamis et al., 2017; Lin et al., 2018). Opinion leaders can provide informal and consumption-related advice to other consumers to assist them in reducing the risks associated with purchasing decisions (Engel et al., 1995; Flynn et al., 1996). Online reviews from reputable or well-known participants have a significant impact on product sales (Chevalier and Mayzlin, 2006). Hence, an increasing number of marketers rely on network celebrities or anchors to persuade e-commerce users to buy (Kim and Kim, 2021; Tafesse and Wood, 2021).

The five main characteristics of Internet celebrities that promote impulse buying are popularity, recognition, homogeneity, social distance, and perceived fit (Li and Du, 2011; Chen et al., 2021), respectively. According to Amazon platform research and analysis, the more famous people are, the more likely they are to have an impact on consumers, resulting in an increase in product sales (Chevalier and Mayzlin, 2006). Consumers' purchase intentions can be influenced by popularity by increasing their emotional trust in anchors (Huffaker, 2010; Edwards et al., 2013). Emotional trust can alleviate hesitancy and expedite the purchasing decision-making process (Chen et al., 2021). Furthermore, the greater the influencer's popularity, the more consumers believe that the products recommended by the influencer have perceived emotional value, resulting in increased purchase intent (Chen et al., 2021). In terms of social media, Kowalczyk and Pounders (2016) reported a positive and significant impact of emotional trust and emotional value to the popularity of traditional celebrities on the likelihood of brand purchase. In general, popularity can influence a consumer's purchase intention both directly and indirectly through emotional trust and perceived emotional value.

The nature of homogeneity is that people tend to imitate others in order to keep up with social trends, which are influenced by values (Kamins, 1990). Consumers tend to imitate Internet celebrities whose values are comparable with their own and purchase the goods they use and recommend (Dittmar et al., 1996). Hence, homogeneous Internet celebrities can directly influence consumers' purchase intention. In addition, Internet celebrities share personal thoughts about products and life, and the presentations seem casual and very real to the audience (Golbeck, 2016). They interact with their followers by liking, commenting, sharing, and retweeting, so users feel that they have a psychological closeness to the online celebrity. Although proximity and reciprocal exchange/relationships are “fictional” (or illusionary) social relationships, consumers are more likely to place emotional trust and emotional value on them. The frequency of live broadcasts, the duration of the live broadcasts, and the viewers' memories (i.e., recall of these exchanges and experiences) reinforce these emotional trust and emotional value. Hence, homogeneity can stimulate consumers' purchase intention through increasing their emotional trust and perceived emotional value (López et al., 2017; Chen et al., 2021). As a result, hypotheses 1 and 2 are proposed:

*Hypothesis 1: As Internet opinion leaders, Internet celebrities' popularity has a positive influence on consumers' purchase intention directly and indirectly through emotional trust or perceived emotional value*.
*Hypothesis 2: As Internet opinion leaders, Internet celebrities' homogeneity has a positive influence on consumers' purchase intention directly and indirectly through emotional trust or perceived emotional value*.

### Atmosphere and Emotional Contagion

Emotions are essentially emotional reactions to personal and social experiences (Du et al., 2011). Individual emotions are influenced by others and their environment in terms of emotional response (Du et al., 2011; Fan et al., 2018; Sun et al., 2019). Customers' emotional responses to the virtual environment of online shopping can influence their behavior (Sun et al., 2019). Furthermore, among consumers who do not interact directly, the emotions of other consumers will influence the emotions and purchase intentions of consumers (Chuah and Yu, 2021). As more conversations and tasks take place in virtual environments, such as e-commerce platforms, emotional contagion can occur without a physical location (in many cases, only through text-based interactions) (Meng et al., 2021). Emotions, according to the existing research, can spread virtually between individuals or groups, as well as more broadly *via* social networking platforms (Peck et al., 2013; Kramer et al., 2014; Del Vicario et al., 2016; Chuah and Yu, 2021; Soderlund et al., 2021). In addition, emotion around the environment could stimulate emotional trust and perceived emotional value, thus stimulating consumers' purchase intention (Pornpitakpan, 2004; Wen et al., 2009; Munasinghe et al., 2019). Therefore, consumers' purchase intention may be influenced by the atmosphere around the live studio on consumers' perceived emotional value and emotional trust. Thus, hypothesis 3 is proposed.

*Hypothesis 3: The atmosphere around the live studio during the live broadcast process has a positive influence on consumers' perceived emotion value and emotional trust, thereby influencing consumers' purchase intention*.

### Promotion and Product

The marketing mix is comprised of the “4Ps” (e.g., product, price, place, and promotion) (Mccarthy and Perreault, 1964; Borden, 1965). As a result of advancements in marketing mix theory, marketing mix has been redefined as a collection of thousands of trace elements gathered together to simplify management activities (Kalyanam and Mcintyre, 2002). Every product evokes a quick, effortless emotional response (Aydinli et al., 2014). Product quality or package is an important factor in directly increasing consumers' purchase intent by influencing consumers' emotional trust and perceived emotional value, thus stimulating consumers' purchase intention (Chinomona et al., 2013; Shaharudin et al., 2013; Hussain et al., 2015; Saleem et al., 2015; Yang et al., 2019).

Promotion should increase the relative impact of emotional value on purchase decisions (Aydinli et al., 2014). Promotions effectively increase consumer emotional trust and perceived emotional value, motivating cosmetics consumers to purchase (Bhatti, 2018; Peng et al., 2019). Therefore, promotion can directly or be based on emotional trust and perceived emotional value to consumers' purchase intention. Therefore, hypotheses 4 and 5 are proposed.

*Hypothesis 4: Promotion positively improves consumers' purchase intention during the live broadcast process, and emotional trust and perceived emotional value play a mediating role*.
*Hypothesis 5: Product positively improves consumers' purchase intention during the live broadcast process, and emotional trust and perceived emotional value play a mediating role*.

**Figure 1:**
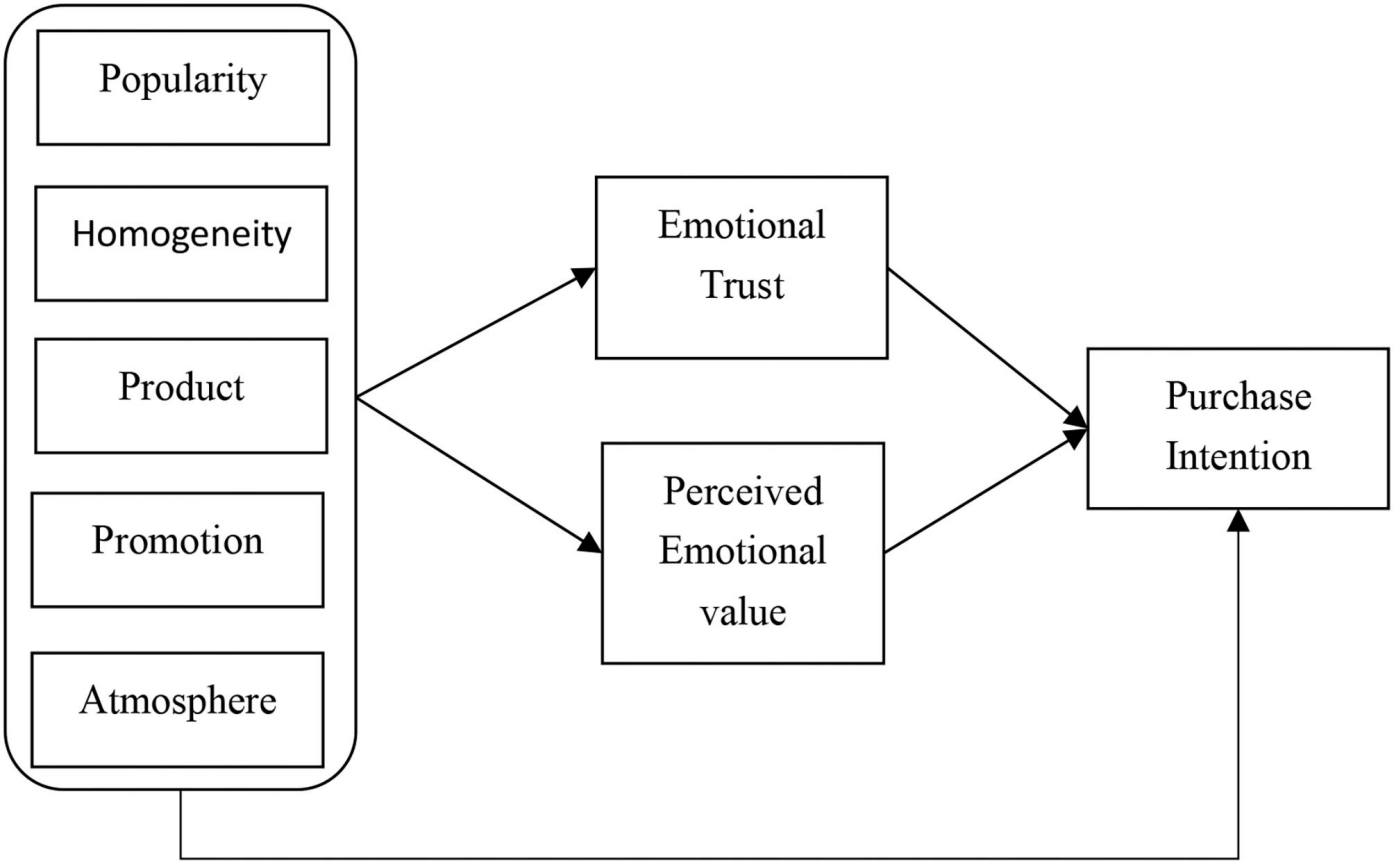
Conceptual model.

Based on the theoretical view, Figure 1 shows the relationships between the constructs and the respective hypotheses.

## Research Design

### Sample Selection and Data Collection

In this study, the cosmetics industry was chosen. A questionnaire was distributed at random by online questionnaire platforms to webcast marketing participants of all genders, ages, incomes, educations, and occupations. For this study, 554 questionnaires were returned, with 154 used in the pilot test to ensure questionnaire rationality and 400 used in the formal test to reveal relationships between variables. The results of an independent sample *t*-test on the individual descriptive statistics of the sample revealed that *p* > 0.05, indicating that there were no significant differences in the covariates (e.g., genders, ages, incomes, educations, and occupations) and that there was no response bias in the questionnaire sample. The scales were all written in English and then translated into Chinese using both forward and reverse translations. (1) In forward translation, the original English scales were translated into Chinese by two Chinese marketing professors, and the Chinese version was created after many iterations of refining items, scoring principles, content, and language. (2) In reverse translation, the Chinese version was translated back into English by two English marketing experts, and this scale was carefully compared, reviewed, and calibrated with the original English scale while being fine-tuned for the Chinese national context and the livestreaming e-commerce situation.

### Measurement Tools

In this study, a more mature scale was used to ensure the questionnaire's reliability and validity, and the scale was modified to account for the survey's context. The specific scale items, as well as relevant references, are listed in Table 1. All eight variables were evaluated using a 5-point Likert scale, with 1 indicating “strongly disagree” and 5 indicating “strongly agree.” To avoid the influence of other control variables, genders, ages, incomes, educations, and occupations were chosen as covariates in this study, with gender and occupations treated as dummy variables and the other variables treated as continuous variables.

**Table 1:** Descriptive statistics of key variable indicators.

**Variables**	**Number**	**Measurement index**	**References**
Popularity (A)	A1	The web celebrity has a certain appeal in the field of cosmetics	Chevalier and Mayzlin, 2006
	A2	The web celebrity has many fans	
	A3	The web celebrity is well-known in the cosmetic field	
Homogeneity (B)	B1	You think the web celebrity is similar to my personality and taste	López et al., 2017
	B2	You think the web celebrity shares my values	
	B3	You think the web celebrity's interest in products is similar to mine	
Product (C)	C1	You will buy cosmetics because the packaging looks good during the live broadcast	Chinomona et al., 2013; Yu et al., 2018; Yeo et al., 2020
	C2	You will buy cosmetics because the web celebrity makeup looks good	
	C3	You think the cosmetics provided by this web celebrity live broadcast are authentic	
Promotion (D)	D1	You think the price of the product recommended by the web celebrity is lower than other channels	Wang and Chen, 2016; Chang, 2017; Bhatti, 2018; Peng et al., 2019
	D2	You buy cosmetics recommended by web celebrity because they are available in trial packs	
	D3	Coupons, gifts or raffles will attract you during the broadcast	
Atmosphere (E)	E1	You are attracted by the web celebrity's sincere emotion and want to buy	Hoonsopon and Puriwat, 2016; Munasinghe et al., 2019; Meng et al., 2021
	E2	You are stimulated by the emotion of your relatives and friends who already bought in the studio	
	E3	You are stimulated by the emotion of other viewers in the studio and want to buy	
Emotional trust (F)	F1	You believe that the information of the product recommended by the web celebrity is true	Koufaris and Hampton-Sosa, 2004; Awad and Ragowsky, 2008
	F2	You believe that the information of the product recommended by the web celebrity is accurate	
	F3	You have confidence in the web celebrity's knowledge and ability about cosmetics	
Perceived emotional value (G)	G1	During the live broadcast you feel delighted	Meng et al., 2021
	G2	During the live broadcast you feel excited	
	G3	During the live broadcast you feel that it is worthy of your learning and recognition	
Purchase intention (H)	H1	You are willing to consider buying cosmetics while watching the live broadcast	Dodds et al., 1991
	H2	You would like to continue to watch the broadcast and consider buying cosmetics	
	H3	You would like to recommend my friends to watch the live broadcast and buy them	

## Data Analysis

### Pilot Test

It is necessary to carry out the pilot test in order to guarantee questionnaire rationality. In the pilot test, 154 valid questionnaires were collected through various channels.

#### Reliability and Validity Analysis

Reliability analysis can verify the reliability of the results and Cronbach's α is used to measure that of the questionnaire. According to previous research, if α is >0.8, it indicates that the data are perfect. If 0.6 < α < 0.8, the data seem fine. If α is <0.6, it proves that the study is bad, that is to say, the data could not be used in the further analysis. The results are shown in Table 2, and it is found that the reliability of all variables was >0.6, which indicated that the scale of the questionnaire is good and is trustworthy.

**Table 2:** Reliability analysis of pilot test.

**Item**	**Cronbach's alpha**	***N***
Popularity	0.749	3
Homogeneity	0.842	3
Product	0.691	3
Promotion	0.690	3
Atmosphere	0.713	3
Trust	0.831	3
Perceived value	0.783	3
Purchase intention	0.838	3

The accuracy of measured results is determined by a validity analysis. There are two types of validity analysis, namely, content validity and structure validity. The structure validity of a questionnaire refers to whether it can theoretically achieve the research goal, whereas the content validity refers to whether it is reasonable based on its content. The questionnaire in this article is based on a number of well-researched studies and has been tailored to the needs of the cosmetics industry. After several revisions, it has strong theoretical support. As a result, the content validity of the questionnaire is high. Typically, exploratory factor analysis (EFA) is used to validate structural validity analysis.

Before conducting EFA, the Kaiser-Meyer-Olkin (KMO) value should be tested using the Bartlett sphere to determine whether the variable is suitable for further factor analysis. When the KMO value approaches one, it indicates that the data are more suitable for factor analysis and that the likelihood of common factors exists is increased. Statistically, a KMO value >0.6 is considered suitable for factor analysis. According to Table 3, all KMO values are >0.6, indicating that all items can perform factor analysis. After factor analysis, the scale was discovered to have eight main factors. According to Table 4, these eight factors, which are all consistent with the original setting, represent popularity, homogeneity, product, promotion, atmosphere, trust, perceived value, and purchase intention, respectively. Each item's factor loading value was >0.7 at the same time, indicating that the scale structure validity was good. To summarize, the reliability and validity tests conducted during the pilot test indicated that the designed questionnaire could be formally investigated.

**Table 3:** KMO and Bartlett's test.

**Item**	**KMO coefficient**	**Significance of Bartlett test**
Web celebrity	0.779	0.000
Product	0.646	0.000
Promotion	0.706	0.000
Atmosphere	0.668	0.000
Trust	0.723	0.000
Perceived value	0.703	0.000
Purchase intention	0.724	0.000

**Table 4:** Rotated component matrix.

	**Component**							
	**1**	**2**	**3**	**4**	**5**	**6**	**7**	**8**
B1	0.867							
B2	0.853							
B3	0.827							
A2		0.833						
A3		0.790						
A1		0.756						
C3			0.833					
C2			0.817					
C1			0.716					
D2				0.805				
D3				0.783				
D1				0.770				
E2					0.847			
E3					0.806			
E1					0.736			
F2						0.870		
F3						0.870		
F1						0.855		
G2							0.851	
G3							0.831	
G1							0.825	
H3								0.880
H2								0.876
H1								0.853

### Formal Test

SPSS version 22.0 is used in this chapter to analyze data from 400 valid questionnaires collected as samples. To begin, descriptive statistical analysis is used to explain the basic information provided by the respondents. To ensure the study's credibility, the reliability and validity of the questionnaire items and data were tested using reliability and validity analysis. Finally, regression analysis and the mediation effect test were used to put the hypothesis to the test.

#### Descriptive Statistics

A descriptive statistical analysis was performed on the demographic variable information of 400 valid samples, and the gender, age, education, income, and occupation were summarized to gain a preliminary understanding of the respondents' basic information distribution.

Table 5 shows that 111 men account for 27.75% of the total, while 289 women account for 72.25%, confirming that women prefer to buy cosmetics over men. When asked if they had purchased cosmetics *via* live broadcast, 283 people said yes (70.75%) and 117 people said no (29.25%), indicating that purchasing cosmetics *via* livestreaming e-commerce has become a popular trend. Cosmetics manufacturers should take advantage of this new channel to increase cosmetics sales. The most common age group, which accounts for 67.5% of the population, is 18–30 years. The second age group, comprising 22.25% of the population, is between the ages of 30 and 45 years. This suggests that young women are most likely to buy cosmetics and most willing to try new things, such as live broadcasting. The highest income levels are 2,000–4,000 yuan and 4,000–6,000 yuan, which are comparable with average wages in China. As a result, cosmetics manufacturers can target a large number of potential customers with a moderate income, and they can create cost-effective cosmetics for them. Undergraduate and college education have the highest rates of completion, with 54.25 and 26.5%, respectively. The sample group is generally well-educated, and they should have a thorough understanding of live broadcast marketing, which will help them complete the second section of the questionnaire. When it comes to occupation, civil servants and private employees have a higher proportion, consistent with their income level. Their purchase intention can also represent plenty of people.

**Table 5:** Demographic characteristics.

**Variables**	**Item**	**Frequency**	**Percent**
Gender	Male	111	27.75%
	Female	289	72.25%
Bought through live broadcast	Yes	283	70.75%
	No	117	29.25%
Age	Under 18	21	5.25%
	18–30	270	67.5%
	30–45	89	22.25%
	Above 45	20	5%
Income	Under 2,000	80	20%
	2,000–4,000	143	35.75%
	4,000–6,000	116	29%
	Above 6,000	61	15.25%
Education	Under high school	21	5.25%
	College	106	26.5%
	Bachelor	217	54.25%
	Above master	56	14%
Occupation	Student	73	18.25%
	Civil servant	144	36%
	Private employee	140	35%
	Freelancer	43	10.75%

#### Reliability and Validity Analysis

According to Table 6, the reliability of all variables was above 0.7, which indicated that the survey scale of this study is good and can be further studied.

**Table 6:** Reliability analysis.

**Name**	**Cronbach's alpha**	***N* of items**
Popularity	0.796	3
Homogeneity	0.828	3
Product	0.748	3
Promotion	0.793	3
Atmosphere	0.733	3
Trust	0.842	3
Perceived value	0.771	3
Purchase intention	0.832	3

According to Table 7, all KMO values are >0.6, indicating that all items can perform factor analysis. After factor analysis, the scale was discovered to have eight main factors. According to Table 8, these eight factors, which are all consistent with the original setting, represent popularity, homogeneity, product, promotion, atmosphere, trust, perceived value, and purchase intention, respectively. Each item's factor loading value was >0.7 at the same time, indicating that the scale structure validity was good.

**Table 7:** KMO and Bartlett's test.

	**KMO coefficient**	**Significance of Bartlett test**
Web celebrity	0.816	0.000
Product	0.691	0.000
Promotion	0.706	0.000
Atmosphere	0.668	0.000
Trust	0.725	0.000
Perceived value	0.694	0.000
Purchase intention	0.722	0.000

**Table 8:** Rotated component matrix.

	**Component**							
	**1**	**2**	**3**	**4**	**5**	**6**	**7**	**8**
B1	0.855							
B3	0.829							
B2	0.803							
A2		0.874						
A1		0.778						
A3		0.752						
C3			0.824					
C1			0.816					
C2			0.809					
D2				0.850				
D3				0.848				
D1				0.824				
E3					0.846			
E2					0.810			
E1					0.765			
F1						0.881		
F3						0.880		
F2						0.854		
G2							0.850	
G3							0.829	
G1							0.805	
H1								0.877
H3								0.863
H2								0.855

#### Regression Analysis

Regression analysis could not only be used to reveal the relations among variables, but also confirm whether the hypothesis could be supported or not. In this section, the independent variables and mediating variables are sequentially added to the model for regression analysis through stepwise analysis. This section analyzes the role of independent variables on the dependent variable and the change in the interpretation of independent variables on the dependent variable when emotional trust and perceived emotional value are used as mediating variables to explore the role of mediating variables. Six regression models on the topic are as follows. *Y*_*i*_ is cosmetic consumers' purchase intention, α_*i*_ is the intercept, β_*i*_ is the independent coefficient, and ε_*i*_ is the residue.

*Model 1: Y*_1_ = α_1_ + β_1_ × *gender* + β_2_ × *age* + β_3_ × *income* + β_4_ × *education* + β_5_ × *occuapation* + ε_1_
*Model 2: Y*_2_ = α_2_ + β_6_ × *popularity* + β_7_ × *homogeneity* + β_8_ × *product* + β_9_ × *promotion* + β_10_ × *atmosphere* + ε_2_
*Model 3: Emotional trust* = α_3_ + β_11_ × *popularity* + β_12_ × *homogeneity* + β_13_ × *product* + β_14_ × *promotion* + β_15_ × *atmosphere* + ε_3_
*Model 4: Y*_4_ = α_4_ + β_16_ × *popularity* + β_17_ × *homogeneity* + β_18_ × *product* + β_19_ × *promotion* + β_20_ × *atmosphere* + β_21_ × *emotional trust* + ε_4_
*Model 5: Perceived emotional value* = α_5_ + β_22_ × *popularity* + β_23_ × *homogeneity* + β_24_ × *product* + β_25_ × *promotion* + β_26_ × *atmosphere* + ε_5_
*Model 6: Y*_6_ = α_6_ + β_27_ × *popularity* + β_28_ × *homogeneity* + β_29_ × *product* + β_30_ × *promotion* + β_31_ × *atmosphere* + β_32_ × *perceived emotional value* + ε_6_

Model 1: Regression of demographic variables and purchase intention

First, demographic variables were regressed to confirm whether gender, age, occupation, education, and income would make contributions to cosmetics consumers' purchase intention.

According to Table 9, apart from age, none of the demographic variables were significant, that is, the consumer's gender, occupation, education, and income characteristics did not exert huge effects on the purchase intention so that the subsequent research could ignore the effect of demographic characteristics on the purchase intention.

**Table 9:** Model 1 regression coefficients and goodness-of-fit tests.

**Model 1**	**Unstandardized coefficients**		**Standardized coefficients**	**T**	***P***
	**B**	**Standard error**	**Beta**		
(Constant)	2.965	0.259		11.427	0.000
Gender	0.117	0.083	0.070	1.412	0.159
Age	0.165	0.060	0.139	2.736	0.007
Income	0.034	0.043	0.044	0.800	0.424
Education	0.087	0.052	0.087	1.658	0.098
Occupation	−0.037	0.046	−0.045	−0.821	0.412
R	0.185				
R^2^	0.034				
Adjusted R^2^	0.022				
Standard estimation error	0.74079				
*F*	2.783				
*P*	0.017

Model 2: Regression of predictive variables and purchase intention

From the results shown in Table 10, we can observe that the model significance is 0.000, which is <0.05, and reaches the significance level. According to the regression results, we found that the significance level of popularity, homogeneity, product, promotion, and atmosphere is <0.05, and the coefficients were 0.122, 0.190, 0.123, 0.256, and 0.214, which shows that the five factors have a significant and positive influence on purchase intention. The promotion has the most effect, while the effect of popularity is the lowest. Meanwhile, atmosphere ranks the second most important. In sum, *Y*_2_ = 0.383 + 0.122 × *popularity* + 0.190 × *homogeneity* + 0.123 × *product* + 0.256 × *promotion* + 0.214 × *atmosphere*.

**Table 10:** Model 2 regression coefficients and goodness-of-fit tests.

**Model 2**	**Unstandardized coefficients**		**Standardized coefficients**	**T**	***P***
	**B**	**Standard error**	**Beta**		
(Constant)	0.383	0.145		2.631	0.009
Popularity	0.122	0.044	0.116	2.751	0.006
Homogeneity	0.190	0.043	0.205	4.465	0.000
Product	0.123	0.055	0.125	2.259	0.024
Promotion	0.256	0.049	0.266	5.195	0.000
Atmosphere	0.214	0.051	0.211	4.199	0.000
R	0.784				
R^2^	0.615				
Adjusted R^2^	0.610				
Standard estimation error	0.46757				
*F*	125.982				
*P*	0.000

Model 3: Regression of predictive variables and intermediate variable as emotional trust.

From Table 11, we can observe that the model significance is 0.000, which is <0.05 and reaches the significance level. According to the regression results, we found that the significance level of homogeneity, product, promotion and atmosphere is <0.05, and the coefficients were 0.142, 0.116, 0.309, 0.330, which shows that the four factors have a significant influence on emotional trust, and the influence is positive. It seems that web celebrity's popularity exerts no effects on consumers' emotional trust while the atmosphere created by anchors during the live broadcast process influences consumers' perceived emotional trust. In sum, *Emotional trust* = 0.237 + 0.142 × *homogeneity* + 0.116 × *product* + 0.309 × *promotion* + 0.330 × *atmosphere*.

**Table 11:** Model 3 regression coefficients and goodness-of-fit tests.

**Model 3**	**Unstandardized coefficients**		**Standardized coefficients**	**T**	***P***
	**B**	**Standard error**	**Beta**		
(Constant)	0.237	0.139		1.697	0.090
Popularity	0.040	0.043	0.037	0.937	0.349
Homogeneity	0.142	0.041	0.150	3.480	0.001
Product	0.116	0.052	0.114	2.207	0.028
Promotion	0.309	0.047	0.314	6.529	0.000
Atmosphere	0.330	0.049	0.318	6.751	0.000
R	0.813				
R^2^	0.661				
Adjusted R^2^	0.657				
Standard estimation error	0.44839				
*F*	153.727				
*P*	0.000

Model 4: Regression of predictive variables, emotional trust and purchase intention

Trust as well as popularity, homogeneity, product, promotion, and atmosphere are simultaneously regressed as independent variables to explore the mediating effect of emotional trust between the independent variables and the purchase intention. The results in Table 12 show that the model fits well and has a significance of 0.000, which is <0.05, indicating that the regression model is significant overall. Among the variables, the significance of trust is 0.000, which has a significant positive effect on purchase intention. The significance of homogeneity and promotion was 0.001 < 0.05 and 0.004 < 0.05, indicating that homogeneity and promotion were still significant after the introduction of the intermediate variable trust, but the coefficients changed from 0.205 to 0.148 and 0.266 to 0.147. The decrease of the coefficients indicates that emotional trust has a partial mediating effect on the two factors of homogeneity and promotion. The significance of product and atmosphere is >0.05, which shows that due to the addition of emotional trust, the two factors that were originally significant no longer have a significant effect on purchase intention. This indicates that trust fully mediates the product and atmosphere factors. Among other variables, the significance of popularity is lower than 0.05. It can be seen that because emotional trust is added into the model, the previous insignificant popularity can become significant, indicating that emotional trust has no mediating effect on popularity. In sum, *Y*_4_ = 0.295 + 0.107 × *popularity* + 0.137 × *homogeneity* + 0.141 × *promotion* + 0.371 × *emotional trust*.

**Table 12:** Model 4 regression coefficients and goodness-of-fit tests.

**Model 4**	**Unstandardized coefficients**		**Standardized coefficients**	**T**	***P***
	**B**	**Standard error**	**Beta**		
(Constant)	0.295	0.137		2.158	0.032
Popularity	0.107	0.042	0.101	2.580	0.010
Homogeneity	0.137	0.040	0.148	3.396	0.001
Product	0.080	0.051	0.081	1.565	0.118
Promotion	0.141	0.049	0.147	2.913	0.004
Atmosphere	0.091	0.050	0.090	1.816	0.070
Emotional trust	0.371	0.049	0.380	7.558	0.000
R	0.815				
R^2^	0.664				
Adjusted R^2^	0.659				
Standard estimation error	0.43745				
*F*	129.462				
*P*	0.000

Model 5: Regression of predictive variables and intervening variable as perceived emotional value.

From Table 13, we can observe that the model significance is 0.000, which is <0.05 and reaches the significance level. According to the regression results, we found that the significance level of homogeneity, product, promotion, and atmosphere is <0.05, and the coefficients were 0.147, 0.238, 0.190, and 0.325, which shows that the four factors have a significant influence on perceived emotional value, and the influence is positive. This indicates that online opinion leaders' popularity does not affect consumers' perceived emotional value, while atmosphere provides the most perceived emotional value for consumers. In sum, *Perceived emotional value* = 0.311 + 0.147 × *homogeneity* + 0.238 × *product* + 0.190 × *promotion* + 0.325 × *atmosphere*.

**Table 13:** Model 5 regression coefficients and goodness-of-fit tests.

**Model 5**	**Unstandardized coefficients**		**Standardized coefficients**	**T**	***P***
	**B**	**Standard error**	**Beta**		
(Constant)	0.311	0.127		2.448	0.015
Popularity	0.011	0.039	0.011	0.292	0.771
Homogeneity	0.147	0.037	0.162	3.944	0.000
Product	0.238	0.048	0.247	4.999	0.000
Promotion	0.190	0.043	0.202	4.415	0.000
Atmosphere	0.325	0.044	0.328	7.311	0.000
R	0.832				
R^2^	0.693				
Adjusted R^2^	0.689				
Standard estimation error	0.40824				
*F*	177.625				
*P*	0.000

Model 6: Regression of predictive variables, perceived emotional value, and purchase intention

Model 6 explores the mediating effect of perceived emotional value between the independent variable and the purchase intention. The results in Table 14 show that the model fit was good and significant at 0.000, which was <0.05, indicating that the regression model was significant overall. Among the variables, the significance of perceived emotional value is 0.000, which has a significant positive effect on purchase intention. The significance of homogeneity and promotion is both <0.05, which indicates that after the introduction of intermediate variable as perceived emotional value, homogeneity and promotion are still significant. However, the decrease of coefficients (0.205–0.136 and 0.266–0.179) suggests that perceived emotional value has a partial intermediary effect on homogeneity and promotion. Since the significance of both product and atmosphere is >0.05, it proves that by adding perceived emotional value into the model, the two previous significant factors are no longer significant, which shows that perceived value has a full mediating effect on product and atmosphere. Among other variables, the significance of popularity is lower than 0.05. Resulting from perceived emotional value, the previous insignificant popularity is significant again, indicating that perceived emotional value has no mediating effect on popularity. In sum, *Y*_6_ = 0.246 + 0, 117 × *popularity* + 0.126 × *homogeneity* + 0.172 × *promotion* + 0.440 × *perceived emotional value*.

**Table 14:** Model 6 regression coefficients and goodness-of-fit tests.

**Model 6**	**Unstandardized coefficients**		**Standardized coefficients**	**T**	***P***
	**B**	**Standard error**	**Beta**		
(Constant)	0.246	0.135		1.815	0.070
Popularity	0.117	0.041	0.111	2.854	0.005
Homogeneity	0.126	0.040	0.136	3.130	0.002
Product	0.019	0.052	0.019	0.356	0.722
Promotion	0.172	0.047	0.179	3.695	0.000
Atmosphere	0.071	0.050	0.070	1.412	0.159
Perceived emotional value	0.440	0.053	0.430	8.248	0.000
R	0.820				
R^2^	0.672				
Adjusted R^2^	0.667				
Standard estimation error	0.43225				
*F*	134.182				
*P*	0.000				

Hence, hypothesis 1 is partially validated, while hypotheses 2–5 are fully validated.

## Discussion

According to Model 1, the influence of demographic characteristics on purchase intention does not need to be considered. Through Model 2, it is proved that anchor's popularity, homogeneity, product, promotion, and atmosphere all have a significant positive effect on consumer's purchase intention but their coefficients are different, which means these variables are not equally important. Atmosphere ranks the second highest at 0.214, indicating that atmosphere based on the theory of emotional contagion is the key variable affecting consumers' purchase intention of livestreaming e-commerce even without the mediating role of emotion, which verifies the effect of emotion in livestreaming e-commerce.

From Models 3 and 5, online opinion leaders' popularity does not affect consumers' emotional trust and perceived emotional value, which identifies that anchors' fame can affect consumers' purchase intention but not influence their emotional trust and perceived emotional value; thereby, hypothesis 1 is partially validated. The specific reason needs to be investigated in further study. However, the atmosphere induced by emotional contagion provides the most emotional trust and perceived emotional value for consumers. Other variables also affect consumers' purchase intention through emotional trust and perceived emotional value.

When emotional trust or perceived emotional value is used as the mediating variable, they have a full mediating effect on product and atmosphere and a partial mediating effect on homogeneity and promotion, which means that product and atmosphere must stimulate consumers' purchase intention through increasing their emotional trust and perceived emotional value, while consumers' purchase intention is affected by homogeneity and promotion directly and indirectly through the intermediary variable as emotional trust or perceived emotional value.

In terms of online celebrities, previous research has mostly focused on the definition (Khamis et al., 2017), the characteristics (Chen et al., 2021), the identification (Li and Du, 2011), and the Influence of opinion leaders in the field of consumer behavior (Kim and Kim, 2021). Furthermore, a recent opinion leadership study focuses on their characteristics' influence in social commerce (Chen et al., 2021). Ladhari et al. (2020) pointed out the relation among YouTube vloggers' popularity, homophily, and emotional attachment, which is the first to introduce emotional marketing into the research of online opinion leaders on consumers' purchasing intention. Meng et al. (2021) identified that online celebrity could induce the emotion of consumers during the live broadcast, such as pleasure emotion, arousal emotion, emotional trust, and admiration emotion, which studied the specific type of emotion induced, but did not study the relationship between the characteristics of Internet celebrities and emotions. Hence, this study is the first to take the characteristics of online opinion leaders as independent variables and emotional trust and perceived emotional value as intermediary variables to study how online opinion leaders influence consumers' purchase intention through the effect of emotion in livestreaming e-commerce and shows that online opinion leaders' popularity and homogeneity could affect consumers' purchase intention directly and online celebrity's homogeneity could influence consumers' purchase intention indirectly through the partial mediating role of emotional trust and perceived emotional value but popularity could not.

With regard to product and promotion, the existing studies are classified as the definition (Kalyanam and Mcintyre, 2002), the influence (Jobber, 2016), the extension (Ronald, 1999), and the application (Mahmoud, 2018). These studies verified that product and promotion could stimulate consumers' purchase intention but did not study the relation between product or promotion and emotion. Some researchers identified the relation between promotion and emotion. Promotion could directly affect consumers' purchase intention and indirectly exert influence through the mediating role of positive emotion (Andani and Wahyono, 2018). Different promotions could induce different emotional perceptions, and it can be a negative emotion, such as the sense of embarrassment or impatience (Yang and Lee, 2016). In short, promotion can stimulate buying through inducing emotions. Aydinli et al. (2014) pointed out that promotion could stimulate emotion, thus influencing purchase decision, which systematically expounded that promotion enhances the role of emotion and reduces the role of reason, but did not make product and promotion as independent variables and emotional trust and perceived emotional value as intervening variables to study how they use the effect of emotion to influence consumers' purchase intention in livestreaming e-commerce. This study fills the research blank and verifies that the influence of product and promotion can be exerted on consumers' purchase intention directly or based on the intermediating variables—emotional trust and perceived emotional value.

As for emotional contagion, current studies are related to the definition (Barsade et al., 2018), the study on mental health (Ni et al., 2020), and the application in the field of offline marketing (Chuah and Yu, 2021). Actually, there are few literatures on the theory of emotional contagion in the field of marketing, and the latest research verifies the impact of online celebrity in livestreaming e-commerce on purchase intention from the perspective of emotional contagion (Meng et al., 2021) but it only studies the influence of specific emotional types of Internet celebrities and other audiences on consumers' purchase intention. The study, for the first time, takes emotional contagion as an independent variable and emotional trust and perceived emotional value as intermediary variables to study how emotional contagion in the live studio affects consumers' purchase intention through the effect of emotion in livestreaming e-commerce.

## Conclusion, Implication, and Limitation

### Research Conclusion

This study investigates how emotion plays a role in affecting consumers' purchase intention during live broadcasts. Through SPSS and linear multiple regression models, it is examined and verified that emotional trust and perceived emotional value can be regarded as mediating variables to stimulate consumers' purchase intention in livestreaming e-commerce. They have a full mediating effect on product and atmosphere and a partial mediating effect on homogeneity and promotion, which identifies that online celebrity's homogeneity and sales promotion can influence consumers' purchase intention through the partial mediating role of emotional trust and perceived emotional value, while product and atmosphere induced by emotional contagion can exert influence on consumers' purchase intention through the full mediating effect of emotional trust and perceived emotional value.

### Theoretical Contributions

Nowadays, there are many literatures on emotional marketing and livestreaming e-commerce. The literature on emotional marketing has been listed in the part of literature review, which shows the research results on emotion in consumer behavior. Livestreaming e-commerce is a novel model of e-commerce, and current studies focus on the role of emotion in livestreaming e-commerce. Lin et al. (2021) examined the role of emotion in interactive and dynamic business settings, such as livestreaming, and identified emotional communication and influence between broadcasters and viewers. Meng et al. (2021) explored the impact of online celebrity in livestreaming e-commerce on purchase intention from the perspective of emotional contagion. Bharadwaj et al. (2022) assessed the sales impact of emotional displays of broadcasters based on a new livestream retail analytics framework. These researchers have studied the role of emotion in the livestream but do not study the mediating effect of emotion and how other factors besides broadcasters affect consumers' purchase intention through emotion. Furthermore, even if the emotional communication between broadcasters and audiences is studied, how the personal characteristics of broadcasters affect consumers' purchase intention through emotion is not studied. These are the research blanks. Therefore, this study is the first to use emotional trust and perceived emotional value as mediating variables to study the effect of emotion on consumers' purchase intention in livestreaming e-commerce. In addition, this study is the first to take the characteristics of online opinion leaders, product and promotion, as well as atmosphere induced by emotional contagion as independent variables, plus emotional trust and perceived emotional value as intermediary variables, to study how these variables influence consumers' purchase intention through the effect of emotions in livestreaming e-commerce.

### Practical Contributions

In terms of opinion leader's homogeneity, livestreaming e-commerce web celebrities should imitate their peers in social commerce to decrease social distance and increase homogeneity with their viewers through communicating with the audience sincerely and considering the audience's needs, thus inducing consumers' emotional trust and perceived emotional value. With regard to promotion, anchors should adopt plenty of preferential activities, such as free trial packs, special offers, coupons, and gifts to stimulate consumers' positive emotions, such as pleasure and emotional trust, thus purchasing during the live broadcast. As for the emotional contagion effect, anchors should pay attention to guiding the live broadcast atmosphere, such as presenting products in a sincere and professional manner and encouraging other purchasers to leave bullet screen comments so as to stimulate consumers' emotional trust and perceived emotional value.

Consumers should purchase goods based on reason, not emotion. For cosmetics business, they should increase their investment in livestreaming e-commerce for its high interaction and a high conversion rate but they should clearly realize that livestreaming can only stimulate consumers' emotion. Improving product quality and after-sales service shall be the key rather than relying on emotional marketing during livestreaming e-commerce.

### Research Limitations and Future Directions

This study contains some limitations. First, the survey conducted by questionnaire can lead to sample selection bias and affect the reliability of the sample data so that advanced measurement tools, such as a PYTHON crawler and text analysis, will be used in future research to draw more accurate conclusions. Furthermore, this study only chooses cosmetics as a research subject and considers insufficiently the difference between product types so that other product types should be investigated and relevant conclusions should be compared in the further research. Finally, we have only studied the influence of positive emotion on consumers' purchasing behavior, and have ignored the role of negative emotion, such as fear, anger, sadness, and so forth. Previous studies on emotions have found that negative emotion may influence consumers' participation in compensatory purchases, and subsequent studies could further explore the mediating effects of negative emotion during livestreams.

## Data Availability Statement

The original contributions presented in the study are included in the article/supplementary material, further inquiries can be directed to the corresponding author.

## Ethics Statement

The studies involving human participants were reviewed and approved by the Committee on Research Ethics of University of Liverpool. Written informed consent to participate in this study was provided by the participants' legal guardian/next of kin.

## Author Contributions

RZ contributed to the conception, the design of the study, and wrote the draft of the manuscript. LT contributed to manuscript revision, read, and approved the submitted version. All authors contributed to the article and approved the submitted version.

## Funding

This study was supported by China University Industry-University-Research Innovation Fund (No. 2020HYB08002) and the Funding of the Research on Regional Cross-border E-commerce Information Service Platform (No. 2021KYCXY002).

## Conflict of Interest

The authors declare that the research was conducted in the absence of any commercial or financial relationships that could be construed as a potential conflict of interest.

## Publisher's Note

All claims expressed in this article are solely those of the authors and do not necessarily represent those of their affiliated organizations, or those of the publisher, the editors and the reviewers. Any product that may be evaluated in this article, or claim that may be made by its manufacturer, is not guaranteed or endorsed by the publisher.
